# Chemotherapy-driven increases in the CDKN1A/PTN/PTPRZ1 axis promote chemoresistance by activating the NF-κB pathway in breast cancer cells

**DOI:** 10.1186/s12964-018-0304-4

**Published:** 2018-11-29

**Authors:** Peng Huang, Deng-jie Ouyang, Shi Chang, Mo-yun Li, Lun Li, Qian-ying Li, Rong Zeng, Qiong-yan Zou, Juan Su, Piao Zhao, Lei Pei, Wen-jun Yi

**Affiliations:** 1 Department of General Surgery, the Second Xiangya Hospital, Central South University, No.139 Renmin Road, Changsha, 410011 China; 2 Department of General Surgery, Xiangya Hospital, Central South University, No.87 Xiangya Road, Changsha, 410008 China

**Keywords:** Breast cancer, Protein tyrosine phosphatase receptor Z1, Pleiotrophin, Chemotherapy resistance, NF-κB signalling pathway

## Abstract

**Background:**

Chemotherapy is the primary established systemic treatment for patients with breast cancer, especially those with the triple-negative subtype. Simultaneously, the resistance of triple-negative breast cancer (TNBC) to chemotherapy remains a major clinical problem. Our previous study demonstrated that the expression levels of PTN and its receptor PTPRZ1 were upregulated in recurrent TNBC tissue after chemotherapy, and this increase was closely related to poor prognosis in those patients. However, the mechanism and function of chemotherapy-driven increases in PTN/PTPRZ1 expression are still unclear.

**Methods:**

We compared the expression of PTN and PTPRZ1 between normal breast and cancer tissues as well as before and after chemotherapy in cancer tissue using the microarray analysis data from the GEPIA database and GEO database. The role of chemotherapy-driven increases in PTN/PTPRZ1 expression was examined with a CCK-8 assay, colony formation efficiency assay and apoptosis analysis with TNBC cells. The potential upstream pathways involved in the chemotherapy-driven increases in PTN/PTPRZ1 expression in TNBC cells were explored using microarray analysis, and the downstream mechanism was dissected with siRNA.

**Results:**

We demonstrated that the expression of PTN and PTPRZ1 was upregulated by chemotherapy, and this change in expression decreased chemosensitivity by promoting tumour proliferation and inhibiting apoptosis. CDKN1A was the critical switch that regulated the expression of PTN/PTPRZ1 in TNBC cells receiving chemotherapy. We further demonstrated that the mechanism of chemoresistance by chemotherapy-driven increases in the CDKN1A/PTN/PTPRZ1 axis depended on the NF-κB pathway.

**Conclusions:**

Our studies indicated that chemotherapy-driven increases in the CDKN1A/PTN/PTPRZ1 axis play a critical role in chemoresistance, which suggests a novel strategy to enhance chemosensitivity in breast cancer cells, especially in those of the triple-negative subtype.

## Background

Breast cancer is the most common malignancy in women and a leading cause of cancer-related death worldwide, while triple-negative breast cancer (TNBC) is a subtype of breast cancer that typically has a relatively poorer outcome compared with other breast cancer subtypes [1, 2]. The TNBC classification applies to all tumours that lack the expression of the endocrine receptors for oestrogen and progesterone (ER and PgR, respectively) and the aberrant expression of HER2. In addition, TNBC typically has an inherently aggressive clinical behaviour and lacks recognized molecular targets for therapy [3]. Nevertheless, chemotherapy is still the most common treatment option for patients with early-stage or advanced-stage TNBC [4]. Approximately 30% of patients with high-grade TNBC have a strong initial response to neoadjuvant chemotherapy (NAC) and achieve a pathologic complete response (pCR); however, the resistance of TNBC to chemotherapy remains a major clinical problem since approximately 20% of patients with TNBC exhibit progression during NAC or shortly after therapy due to drug resistance [5]. Therefore, understanding the molecular basis of chemotherapy and identifying novel molecular targets are essential to improving chemotherapy efficacy in patients with TNBC.

Recently, multiple lines of evidence suggested that the expression of pleiotrophin (PTN), which is a secreted growth factor that binds to receptor protein tyrosine phosphatase zeta (PTPRZ1) to stimulate human endothelial cell migration, is associated with poor prognosis in a variety of malignant tumours [6]. Our previous study found that variation in the expression of PTPRZ1 was observed between recurrent TNBC tissue and nonrecurrent TNBC tissue, which indicated that PTPRZ1 may be a novel risk factor for poor prognosis in TNBC [7]. However, the precise mechanisms whereby PTN/PTPRZ1 signalling regulates the chemotherapy sensitivity of TNBC cells are not well understood.

Our current studies showed that the expression of PTN and its receptor PTPRZ1 in human breast cancer tissue depended on whether the patient received chemotherapy, and chemotherapy-driven increases in PTN/PTPRZ1 signalling could inhibit chemotherapy responsiveness in TNBC cells. In the current work, mechanistic and functional studies were performed to thoroughly elucidate the molecular players involved in the PTN/PTPRZ1 signalling that regulates chemoresistance and to determine the potential roles of PTN/PTPRZ1 in chemotherapy resistance.

## Methods

### Microarray analysis of data from the GEPIA database and GEO database

To investigate the expression levels of the same gene in breast cancer and normal tissues, we performed an analysis using the GEPIA database (Gene Expression Profiling Interactive Analysis, http://gepia.cancer-pku.cn/index.html) [8], which contains 1085 breast cancer tissue samples and 291 normal tissue samples. To compare the expression levels of the same gene in breast cancer tissue before and after chemotherapy, we analysed a cohort from the GEO database (GSE87455, https://www.ncbi.nlm.nih.gov/geo/) that included 69 samples from breast cancer patients with gene expression data (pretreatment/posttreatment).

### Cell culture

The breast cancer cell lines HCC-1937, MCF-7, MCF-7/ADR, MDA-MB-231 and MDA-MB-453 were purchased from the Cell Bank of the Chinese Academy of Sciences (Shanghai, China). The cells were grown in 25 cm^2^ cell culture flasks and maintained in Dulbecco’s-modified Eagle’s medium (DMEM, HyClone) or RPMI 1640 medium (HyClone) supplemented with 10% foetal bovine serum, 100 U/ml penicillin G, and 100 U/ml streptomycin at 37 °C in 5% CO2 and 95% air as previously described [9]. Dox was purchased from Yuanye Biotech (Shanghai, China) and diluted in PBS. rhPTN was purchased from PeproTech (London, UK), and cells were treated with rhPTN (5 μg/mL) for 20 min according to the manufacturer’s instructions.

### Immunofluorescent staining

Cells were washed with PBS twice and then fixed with 4% paraformaldehyde. The cells were incubated with anti-PTPRZ1 primary antibodies (BD Biosciences, 1:200) overnight at 4 °C. Cells were washed with PBS and then incubated with the appropriate conjugated secondary antibodies (Sangon Biotech, Shanghai, China, 1:500) for 1 h at 37 °C. Finally, the cells were stained with DAPI (50 μg/ml) for 20 min for nuclear imaging. At least 100 cells per well were counted in 3 independent experiments.

### Quantitative RT-PCR

Total RNA was extracted from breast cancer cells using Trizol (Invitrogen, Carlsbad, CA). As described in our previous work [9], qRT-PCR was performed on a Prism® 7300 Real-time PCR System (Applied Biosystems, Foster City, CA, USA) using a SYBR® Green PCR master mix (Applied Biosystems, Foster City, CA, USA). After the reactions were completed, the comparative threshold cycle (Ct) method was used to calculate the relative gene (e.g., PTN, PTPRZ1, IKKα, IKKβ, IκBα, p65 and p50) expression. U6 expression was used as the internal control. Human-specific primers were synthesized, and their sequences are shown in Table 1.

**Table 1:** Primers designed for RT-PCR

Gene		Sequence
PTPRZ1	Sense	TGGGAAAACAGTGGAAAT
	Antisense	CCGCATCAAAGCAGTAGA
β-actin	Sense	AGGGGCCGGACTCGTCATACT
	Antisense	GGCGGCACCACCATGTACCCT
IKKα	Sense	TTGTAGTTTAGTAGTAGAACCCAT
	Antisense	ATTCCAGTTTCACGCTCA
IKKβ	Sense	TGAATGAGGATGAGAAGACTG
	Antisense	GACCACGGACCTTGCTAC
IκBα	Sense	GCAGCAGACTCCACTCCAC
	Antisense	TCCACGATGCCCAGGTAG
p65	Sense	GGCCATGGACGAACTGTT
	Anti-sense	GGTCTTGGTGGTATCTGTGCT
p50	Sense	GTCTTACCCTCAGGTCAAAA
	Antisense	TGTCATTCGTGCTTCCAG

### Cell transfection

The PTPRZ1-overexpression plasmid and CDKN1A-overexpression plasmid were obtained from Professor Jun-li Luo (Laboratory of Gene Regulation and Signal Transduction, Department of Pharmacology and Cancer Center, School of Medicine, UCSD, La Jolla, California, USA) as gifts, and the knockdown plasmids were constructed by GenePharma (GenePharma Co., Ltd., Shanghai, China). The sequences of siPTPRZ1 (sense: 5′-AAAUGCGAAUCCUAAAGCGUU-3′; antisense: 5′-AACGCUUUAGGAUUCGCAUUU-3′), siPTN (sense: 5′-CCAGCAAUAUCAGCAGCAATT; antisense: 5′-UUGCUGCUGAUAUUGCUGGTT), and siCDKN1A (sense: 5’-GAUGGAACUUCGACUUUGUTT-3′; antisense: 5’-ACAAAGUCGAAGUUCCAUCTT-3′) were the same as those in previous reports [10, 11]. Lipofectamine and Plus reagent (Invitrogen) was used for transfection following the manufacturer’s instructions. After incubation with the transfection mixture for 6–8 h, cells were cultured with fresh medium containing 10% FBS for the following experiments.

### Western blot analysis

Nuclear and cytoplasmic proteins were fractionated as previously described [12]. The quantification of the protein extract was carried out using a BCA protein quantitation kit (P001B, Auragene, Changsha, China). The protein samples (25 μg) were fractionated on 8% SDS-polyacrylamide gels and transferred onto pure nitrocellulose membranes (Sangon Biotech, Shanghai, China). The membranes were then probed with primary antibodies, and GAPDH and H1 were used as internal controls. The following primary antibodies were used for western blotting: anti-PTN (Abcam, #ab79411, 1:10000); anti-PTPRZ1 (Abcam, #ab126497, 1:1000); anti-CDKN1A (Santa Cruz, #sc-817, 1:1000); anti-p-IKKα/β (Ser176/180) (Cell Signaling, #2697S, 1:1000); anti-IKKα (Cell Signaling, #2682S, 1:1000); anti-IKKβ (Cell Signaling, #2684S, 1:1000); anti-p-IκBα (Cell Signaling, #2859S, 1:1000); anti-IκBα (Cell Signaling, #9242S, 1:1000); anti-p-p65 (Ser536) (Cell Signaling, #3031S, 1:1000); anti-p65 (Cell Signaling, #8242S, 1:1000); anti-H1 (Santa Cruz, #sc-8030, 1:1000); and anti-GAPDH (Abcam, #ab9485, 1:2500).

### Colony formation efficiency assay

After treatment, MDA-MB-231 cells were plated in a six-well plate at a density of 300 cells/well and the six-well plate was incubated at 37 °C in a humidified incubator for 10 days. The cell colonies were stained with 0.5% crystal violet solution (Solarbio, Beijing, China), and the colonies were counted.

### Cell proliferation assay

The in vitro growth of cells treated with Dox (Yuanye Biotech, Shanghai, China) or Cisplatin (DDP, Yuanye Biotech, Shanghai, China) was detected with a CCK8 assay kit (Sangon Biotech, Shanghai, China) according to the manufacturer’s instructions. The cell growth curve was generated from the corresponding normalized OD450 values.

### Flow cytometry analysis

The cells for the apoptosis analysis were stained with anti-Annexin V antibody and propidium iodide using an Annexin V-FITC Apoptosis Detection kit (Sangon Biotech, Shanghai, China), and the percentage of apoptotic cells was examined with flow cytometry (BD Bioscience, CA, USA).

### Microarray analysis

Microarray analysis was conducted to investigate the expression changes of chemotherapy-driven genes. MDA-MB-231 cells were treated with Dox (1 μg/ml) or PBS for 24 h in triplicate, and then equal quantities of the cells from each triplicate were mixed to generate one sample for each group. Microarray-based mRNA expression profiling was carried out at Genenergy Biotech (Shanghai, China).

### Statistical analyses

All data were included for statistical analyses performed with SPSS 22.0 or GraphPad Prism 6.0 software. An unpaired Student’s t-test (two-tailed) was used for the comparison between two unpaired groups, and one-way ANOVA was applied for multi-group data comparison. Bar graphs are presented as the mean ± s.e.m., with the level for statistical significance set to *p < 0.05 or **p < 0.01.

## Results

### The expression of PTN and its receptor PTPRZ1 were upregulated in human breast cancer tissue after chemotherapy

**Fig. 1:**
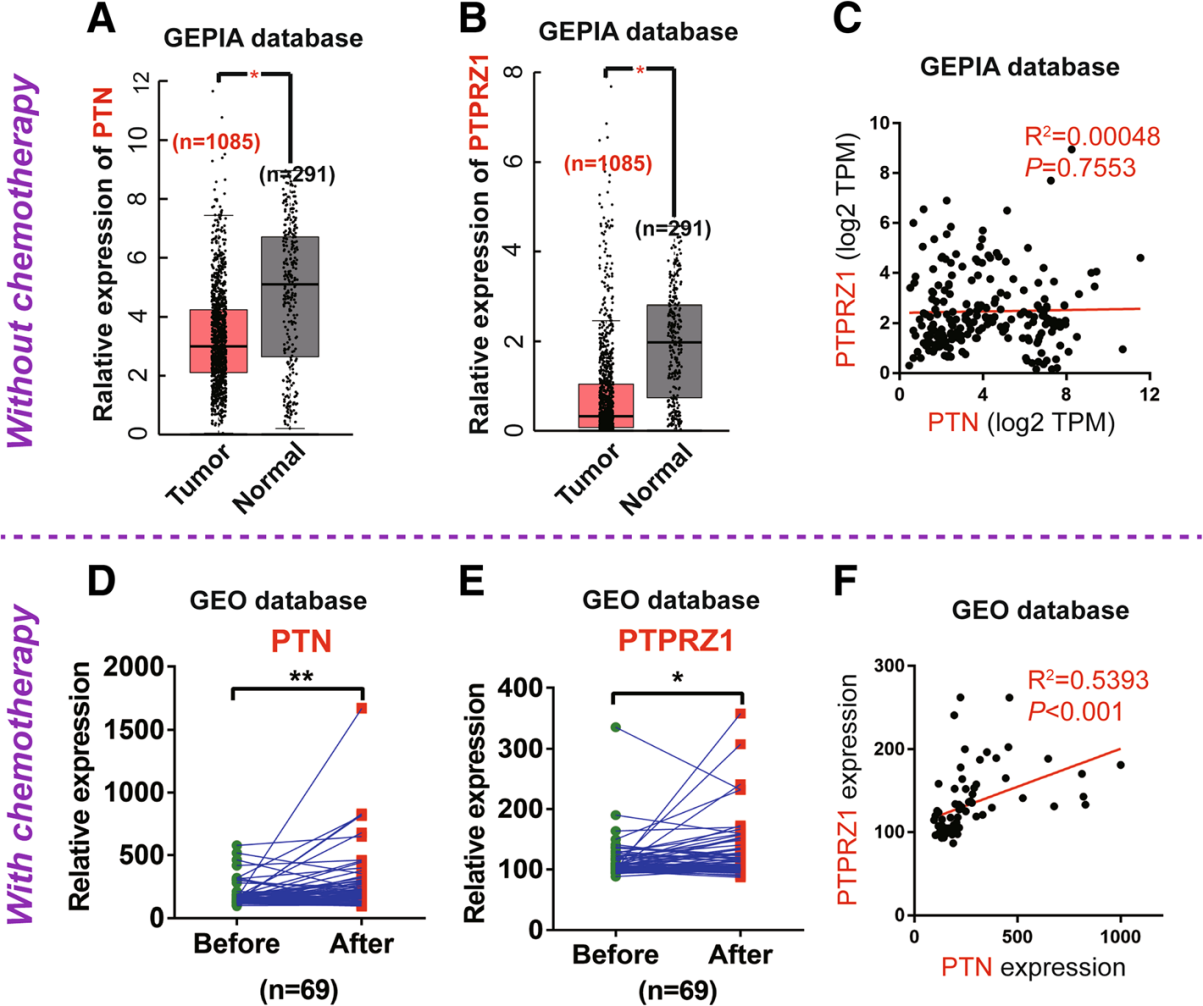
The expression levels of PTN and PTPRZ1 were increased in breast cancer tissue after chemotherapy. PTN (**a**) and PTPRZ1 (**b**) expression data in human normal breast tissue (*n* = 291) and breast cancer tissue (*n* = 1085) from breast cancer patients without chemotherapy were obtained from the GEPIA database (http://gepia.cancer-pku.cn/index.html), Student’s t-test, *p < 0.05. **c** The correlation analyses of PTN and PTPRZ1 expression in breast cancer specimens without chemotherapy from the GEPIA database are shown, n = 1085, *p* = 0.7557, Pearson’s r test. Paired expression data of PTN (**d**) and PTPRZ1 (**e**) in human breast cancer specimens before and after adjuvant chemotherapy were obtained from the GEO database (https://www.ncbi.nlm.nih.gov/geo/), *n* = 69, t-test, *p < 0.05, **p < 0.01. **f** The correlation analyses of PTN and PTPRZ1 expression in breast cancer specimens after adjuvant chemotherapy is shown, n = 69, *p* < 0.001, Pearson’s r test

To assess the expression of PTN and PTPRZ1 in breast cancer and their relationship with chemotherapy, we obtained RNA sequencing expression data for 291 normal breast tissue samples and 1085 cancer tissue samples from the GEPIA database (http://gepia.cancer-pku.cn/index.html); none of the patients who donated the samples were treated with chemotherapy; additionally, we downloaded matched before and after chemotherapy gene expression data for breast cancer tissue samples from 69 patients from the GEO database (GSE87455) (https://www.ncbi.nlm.nih.gov/geo/). The expression levels of both PTN and PTPRZ1 were significantly downregulated in the breast cancer group compared to the normal group (*p* < 0.05) (Fig. 1a, b), and there was no significant correlation between the expression levels of PTN and PTPRZ1 (*p* > 0.05, R^2^ = 0.00048) (Fig. 1c). Interestingly, the expression levels of PTN and PTPRZ1 were both upregulated after chemotherapy (Fig. 1d, e), and a significantly positive correlation between the expression levels of the two proteins was also observed (*p* < 0.05, R^2^ = 0.5393) (Fig. 1f).

### Doxorubicin upregulated the expression of PTN and PTPRZ1 and formed a positive feedback loop in TNBC cells

**Fig. 2:**
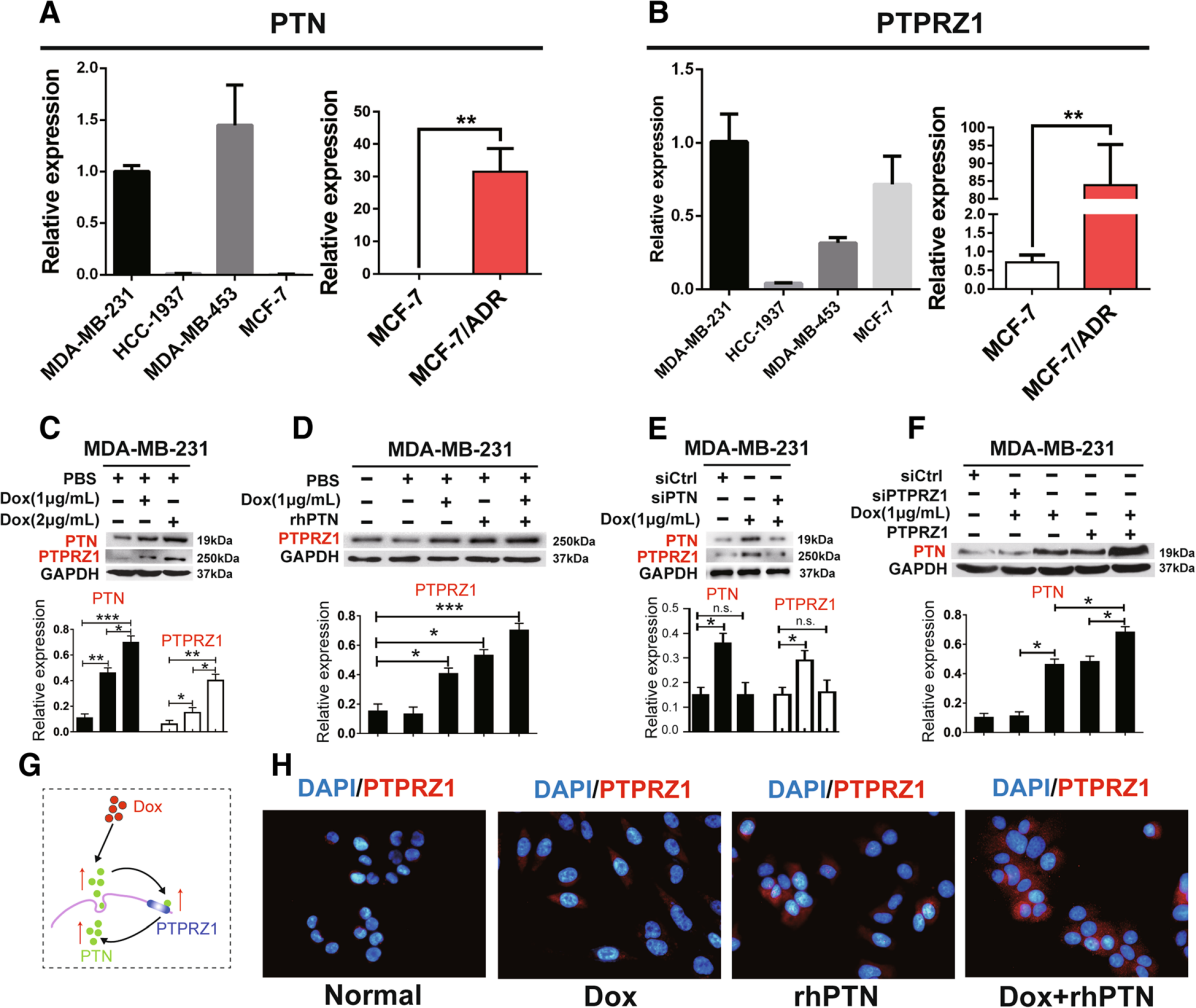
Dox upregulated the expression of PTN and its receptor PTPRZ1 in breast cancer cells. **a**, **b** RT-PCR analyses of the expression of PTN and PTPRZ1 in the MCF-7/ADR, MCF-7, MDA-MB-231, HCC-1937, and MDA-MB-453 cell lines. The data are shown as the mean ± s.e.m., *n* = 5, **p < 0.01, Student’s t-test. Immunoblot analyses of the expression of PTN and PTPRZ1 in MDA-MB-231 cells treated with different concentrations of Dox (**c**), rhPTN (**d**), scrambled siRNA (siCtrl) or empty vector plasmid (vector) (**e**), and PTPRZ1 knockdown (siRNA) or overexpression (PTPRZ1) were performed. (**f**). The representative blots are shown in the upper panel, and the summary densitometry measurements are shown in the lower panel. Data are shown as the mean ± s.e.m., n = 5, *p < 0.05, ***p < 0.001, Student’s t-test. **g** The chemotherapy-driven increases in PTN and PTPRZ1 expression levels create a positive feedback loop. **h** Immunofluorescent staining for PTPRZ1 (in red) in MDA-MB-231 cells treated with Dox, rhPTN or Dox + rhPTN is shown

We then detected the expression of PTN and PTPRZ1 in different breast cancer cell lines via RT-PCR, and we found that PTN and PTPRZ1 were expressed at higher levels in the MDA-MB-231 and MDA-MB-453 TNBC cells than the other breast cancer cells, HCC-1937 and MCF-7. We also found that the expression levels of PTN and PTPRZ1 were significantly increased in chemotherapy-resistant cell lines (MCF-7/ADR) compared with those of the chemotherapy-sensitive cell line (MCF-7) (Fig. 2a, b). Dox treatment increased the expression of PTN and PTPRZ1 in MDA-MB-231 TNBC cells in a dose-dependent manner (Fig. 2c). Additionally, in MDA-MB-231 cells, we found that Dox and PTN are two independent factors that promote the upregulation of PTPRZ1 expression (Fig. 2d, h). To address whether PTPRZ1 also affected the expression of PTN, we investigated whether knockdown or increasing PTPRZ1 expression in MDA-MB-231 cells could attenuate or enhance the Dox-driven PTN upregulation. We found that decreased PTPRZ1 expression could significantly offset the Dox-driven increase in the PTN level in MDA-MB-231 cells; conversely, when the expression of PTPRZ1 increased, the Dox-driven PTN level correspondingly increased (Fig. 2f). These data definitively demonstrated that chemotherapy upregulated the expression of PTN and PTPRZ1 in TNBC cells and formed a positive feedback loop between the expression of PTN and that of PTPRZ1 (Fig. 2g).

### PTN/PTPRZ1 reduced chemosensitivity by promoting tumour proliferation and inhibiting apoptosis

**Fig. 3:**
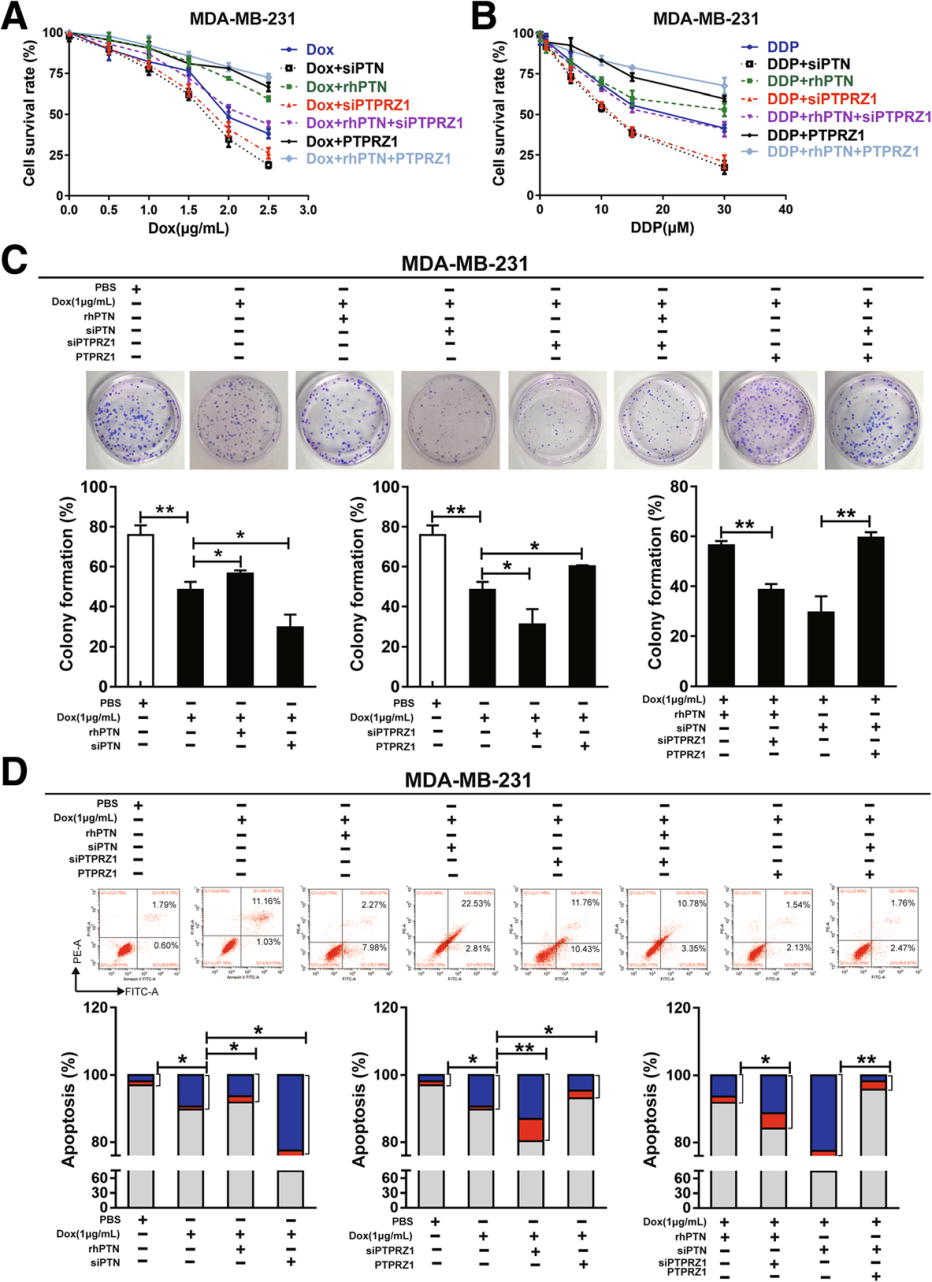
PTN/PTPRZ1 are involved in breast cancer cell chemoresistance. MDA-MB-231 cells, which were transfected with siPTPRZ1, PTPRZ1, or siPTN to regulate endogenous protein, or added exogenous protein (rhPTN) in the cell culture medium to increase PTN levels, were treated with different concentrations of Dox or DDP. **a**, **b** Cell viability was determined with a CCK8 assay. **c** Cell growth was measured by colony formation assays. The data are shown as the mean ± s.e.m. of three independent experiments. The statistical significance of the between-group differences was evaluated with a Student’s t-test, *p < 0.05, **p < 0.01, n.s. means not significant. **d** Apoptosis was measured by flow cytometry based on Annexin V/PI double staining. The data are shown as the mean ± s.e.m. of three independent experiments. The statistical significance of the between-group differences was evaluated with a Student’s t-test, *p < 0.05, **p < 0.01

To understand whether the chemotherapy-driven upregulations of PTN and PTPRZ1 expression levels functioned as tumour protective factors that promote breast cancer cell growth, we investigated whether knockdown or increasing PTN and PTPRZ1 expression could attenuate the effect of chemotherapy in vitro. Our results confirmed that upregulating PTPRZ1 expression or adding rhPTN in the cell culture medium could offset the proliferation inhibition of Dox or DDP, whereas knockdown the expression of PTPRZ1 could enhance the proliferation inhibition of chemotherapy in MDA-MB-231 cells (Fig. 3a, b). Consistently, knockdown of the expression level of PTN or PTPRZ1 significantly increased the inhibition of colony formation (Fig. 3c) and the number of apoptotic cells (Fig. 3d), which correspondingly indicated that siPTN and siPTPRZ1 exerted chemotherapy sensitization effects. Furthermore, we also demonstrated that the inhibition of colony formation and promotion of apoptosis by Dox were rescued when exogenous PTN or endogenous PTPRZ1 was increased (Fig. 3c and d) in MDA-MB-231 cells.

### CDKN1A regulated dox-driven PTN secretion in TNBC cells

**Fig. 4:**
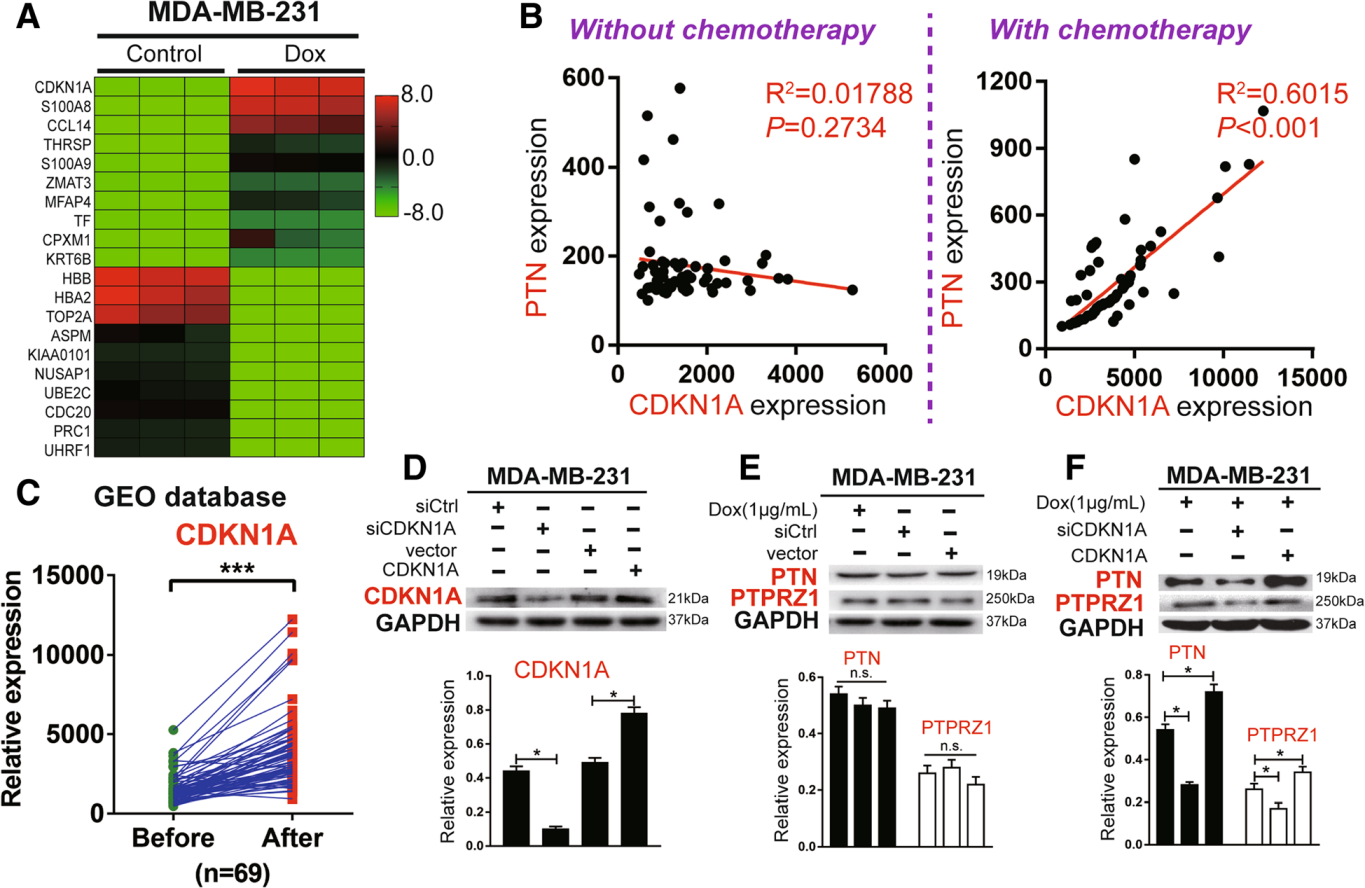
CDKN1A is transcriptionally regulated by Dox and affects the expression of PTN in breast cancer cells. **a** MDA-MB-231 cells were treated with Dox (1 μg/ml) or PBS for 24 h, and a heatmap of the 20 genes that showed the largest changes in gene expression between the two groups determined by microarray analysis is shown. **b** The correlation analyses of PTN and CDKN1A expression in breast cancer specimens with or without chemotherapy from the GEO database are shown, n = 69, Pearson’s r test. **c** The paired expression data of CDKN1A in breast cancer specimens before and after adjuvant chemotherapy were obtained from the GEO database, n = 69, t-test, ***p < 0.001. **d**, **e**, **f** Immunoblot analyses of the expression of CDKN1A, PTN, and PTPRZ1 in MDA-MB-231 cells undergoing treatment with Dox and knockdown or increasing the expression of CDKN1A by siRNA treatment or plasmid overexpression, respectively, are shown. Representative blots are shown in the upper panel, and summary densitometry measurements are shown in the lower panel. The data are shown as the mean ± s.e.m., n = 5, *p < 0.05, n.s. means not significant, Student’s t-test

To explore the potential upstream mechanism that regulates chemotherapy-driven PTN/PTPRZ1, we analysed the Dox-affected genes in TNBC cells with a high-throughput microarray. We noticed that CDKN1A (cyclin-dependent kinase inhibitor 1 A) ranked first among 60 genes significantly upregulated after Dox treatment (average fold changes> 3.0) relative to the matched negative control in MDA-MB-231 cells (Fig. 4a). When we used the same GEO microarray datasets (GSE87455), we found that there was a significant correlation between the expression levels of CDKN1A and PTN after chemotherapy (*p* < 0.05, R^2^ = 0.6015), whereas there was no significant correlation before treatment (*p* > 0.05, R^2^ = 0.01788) (Fig. 4b). Interestingly, we found that the expression of CDKN1A was significantly upregulated after chemotherapy (Fig. 4c). To confirm the relationship between CDKN1A and Dox-driven PTN expression or Dox-driven PTPRZ1 expression, we knocked down or increased the expression of CDKN1A in MDA-MB-231 cells treated with Dox. We found that the expression levels of CDKN1A and Dox-driven PTN or Dox-driven PTPRZ1 formed a positive correlation. Additionally, the regulatory effect of CDKN1A on PTPRZ1 was not as obvious as the effect on PTN, so we presumed that CDKN1A may directly regulate PTN levels, but the present results were not enough to prove whether CDKN1A directly regulates PTPRZ1 levels (Fig. 4e and f).

### Elevated CDKN1A expression correlated with chemoresistance in TNBC cells

**Fig. 5:**
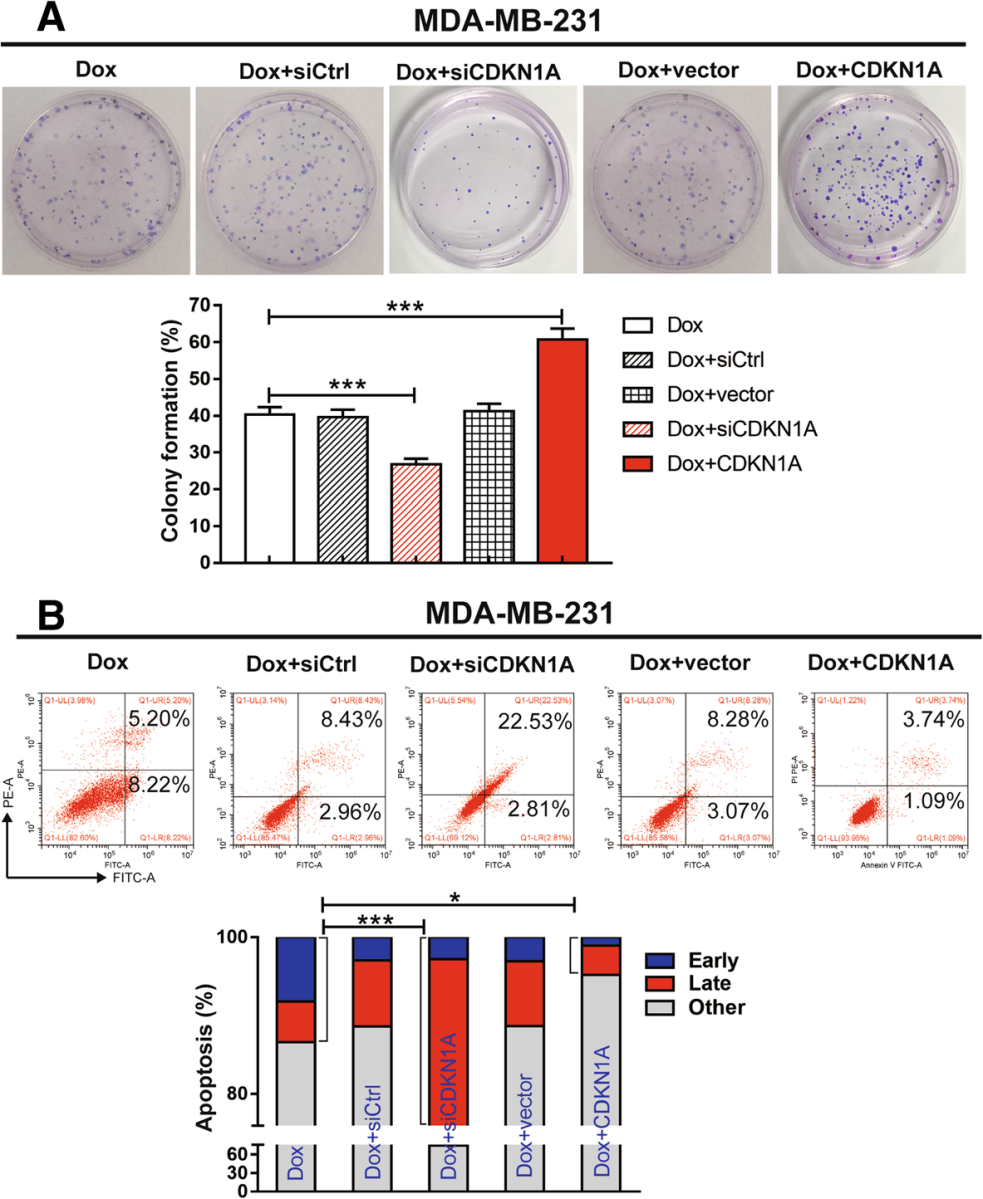
CDKN1A is required for chemoresistance in breast cancer cells. The expression of CDKN1A in MDA-MB-231 cells was knocked down with specific targeting siRNA or overexpressed with a plasmid, and then the cells were treated with Dox (1 μg/ml) for 24 h. **a** Cell growth was measured by colony formation assays. The data are shown as the mean ± s.e.m. of three independent experiments. The statistical significance of the differences between the experimental group and control group (Dox) was evaluated with a Student’s t-test, ***p < 0.001. **b** Apoptosis was measured by flow cytometry based on Annexin V/PI double staining. The data are shown as the mean ± s.e.m. of three independent experiments. The statistical significance of the differences between the experimental group and control group (Dox) was evaluated with a Student’s t-test, *p < 0.05, ***p < 0.001

CDKN1A has been described previously as an inhibitor of cyclin-dependent kinase and plays an important regulatory role in cell proliferation, differentiation and senescence, but its function in the development of different cancers is not consistent [13–15]. To investigate whether CDKN1A acts as a tumour protective factor in TNBC during chemotherapy, we evaluated the effect of knocking down or increasing the expression of CDKN1A on colony formation and apoptosis in MDA-MB-231 cells. We demonstrated that the ability to undergo colony formation or apoptosis under Dox treatment was significantly affected by the change in CDKN1A expression, and CDKN1A expression protected MDA-MB-231 cells from inhibition by Dox (Fig. 5a and b).

### NF-κB pathway activation by excess PTPRZ1

**Fig. 6:**
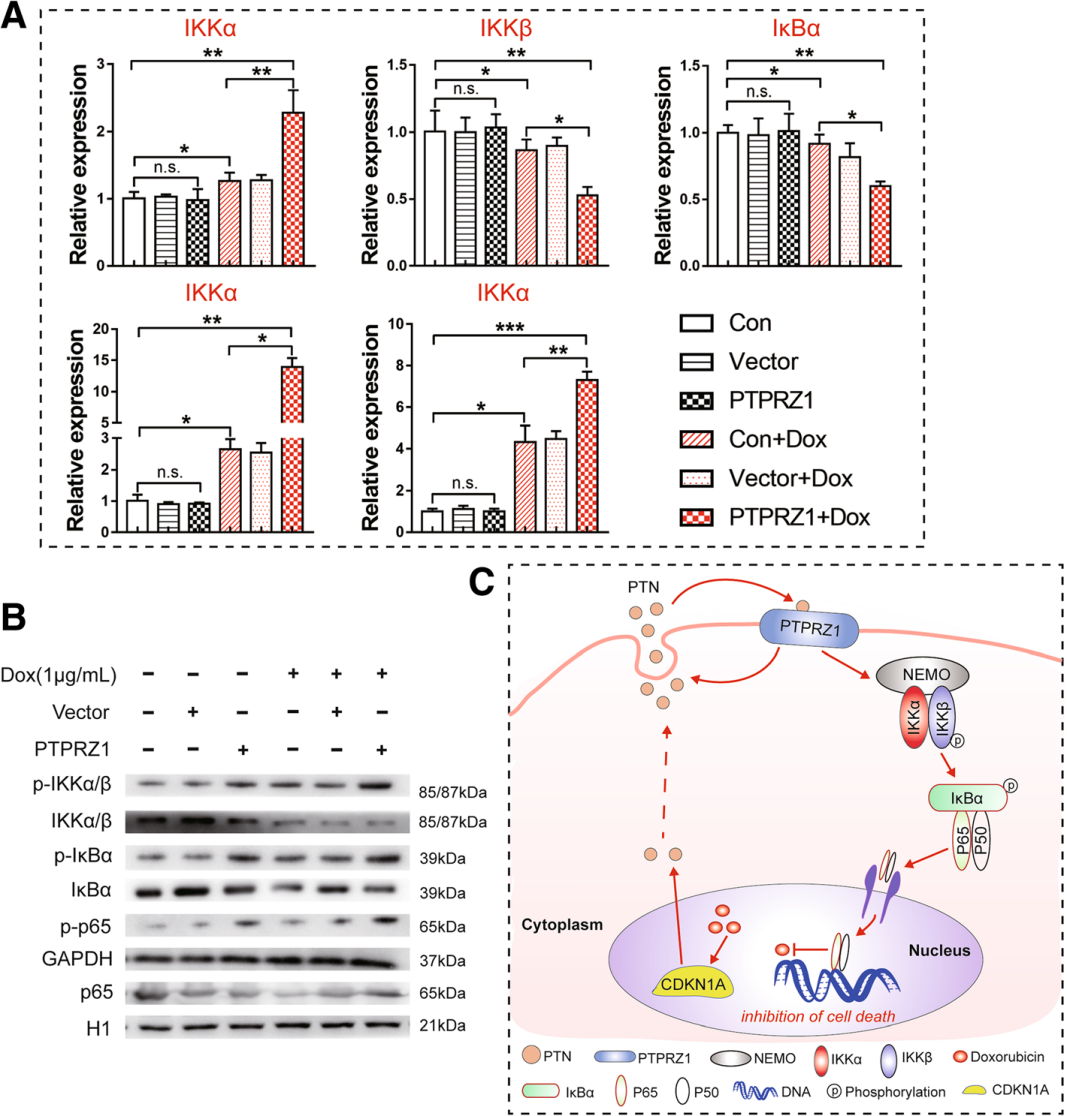
PTPRZ1 activated the NF-κB pathway in breast cancer cells following treatment with Dox. **a** RT-PCR analyses of the expression of NF-κB pathway components, including IKKα, IKKβ, IκBα, p65 and p50, in MDA-MB-231 cells transfected with PTPRZ1, siPTPRZ1 or scrambled siRNA (vector) and then treated with Dox (1 μg/ml) for 24 h are shown, *n* = 3, Student’s t-test, *p < 0.05, **p < 0.01, ***p < 0.001. **b** The phosphorylation of NF-κB pathway components was detected by western blotting; NF-κB activation was investigated by assessing the phosphorylation of IKKα/β (Ser176), IκBα (Ser32) and p65(Ser536). **c** Proposed model for mechanisms of CDKN1A/PTN/PTPRZ1-induced chemoresistance in breast cancer cells

Recently, multiple studies have demonstrated that NF-κB activation enhances chemoresistance processes [16]. Here, we found that the classical NF-κB signalling pathway was activated in TNBC cells by treatment with Dox. The relative expression levels of NF-κB pathway components, such as IKKα, p65 and p50, were upregulated, while the relative expression levels of IKKβ and IκBα were downregulated according to an analysis using RT-PCR (Fig. 6a). We also demonstrated that the gene expression pattern of the Dox + PTPRZ1 group was similar to that of the Dox group, but the relative expression levels of the genes of the NF-κB signalling pathway were significantly higher or lower in the Dox + PTPRZ1 group compared to the vector control group (Fig. 6a). To obtain further proof of NF-κB activation, the phosphorylation of NF-κB pathway components was also detected by western blotting. We found that the levels of phosphorylated NF-κB pathway components, such as p-IKKα/β (Ser176), p-IκBα (Ser32) and p-p65(Ser536), were increased after NF-κB activation, while non-phosphorylated NF-κB components were unaffected or showed a trend towards reduced levels (Fig. 6b). Therefore, our results strongly suggested that chemotherapy-driven PTPRZ1 expression enhanced the activation of the NF-κB signalling pathway during chemoresistance processes in TNBC cells (Fig. 6c).

## Discussion

Chemotherapy plays an important role in breast cancer management, and one of the main barriers to cure breast cancer is the chemoresistance, both intrinsic and therapy-induced, of breast cancer, especially the TNBC subtype [17]. To overcome this obstacle, it is important to identify the critical determinants of chemoresistance and to develop safe and effective tumour chemosensitizers. Recently, emerging evidence has demonstrated that PTN/PTPRZ1 signalling might be involved in the carcinogenesis of several cancers; PTPRZ1 interacts with its ligand PTN to mediate tumour cell growth and metastasis [18]. It has been demonstrated that PTPRZ1 binding to PTN could activate calmodulin to induce nitric oxide formation, which causes tumour blood vessel formation and tumour cell proliferation in vitro [19]. Moreover, downregulating PTPRZ1 expression in glioblastoma multiforme (GBM) cells produced a decrease in tumour migration and proliferation [18]. Similar results have also been documented in the carcinogenesis of breast cancer. On the one hand, studies have found that a low number of breast cancer cases have detectable expression of PTN, while normal breast tissue does not express PTN, while on the other hand, PTN gene expression could play a role in the invasive and/or metastatic behaviour of cell lines or the original tumours the cell lines were obtained from, and targeting PTN with a dominant negative PTN reversed the malignant phenotype of human breast cancer cells in vivo [20, 21].

In recent years, conflicting data on the role of PTN and PTPRZ1 expression in breast cancer was existed in different reports, some studies shown over-expression and others shown decreased expression, what interested us was that over-expression of PTN or PTPRZ1 often related to a more aggressive breast cancer phenotype [22, 23]. Our previous study demonstrated that PTN and PTPRZ1 were highly expressed in recurrent TNBC tissue, which hinted at their tumour-promoting effects [7]. It is well known recurrent TNBC tissues are that kind of breast cancer resistant to chemotherapy and most of them coming from the aggressive phenotype. That means chemotherapy drugs may be one of the activation factors for PTN/PTPRZ1 pathway, following with an even bad chemotherapy sensitivity to these aggressive breast cancer phenotypes. In this study, we uncovered that the protein levels of PTN and PTPRZ1 are significantly higher in human breast cancer tissue after receiving chemotherapy compared with cancer tissue from the same patient prior to chemotherapy. The disruption of PTN/PTPRZ1 signalling by siRNAs could largely abrogate the therapeutic resistance effects of PTN/PTPRZ1 signalling and largely suppress TNBC cell growth during chemotherapy. It is well confirmed that three PTPRZ isoforms are generated by alternative splicing from a single PTPRZ gene: two transmembrane isoforms, PTPRZ-A and PTPRZ-B, and one secretory isoform, PTPRZ-S [24]. Since PTPRZ binds PTN mostly through its glycans, deglycosylation of PTPRZ-B would definitely reduce the affinity of PTPRZ-B for PTN [25]. In this study, only the deglycosylated form of PTPRZ-B was observed in breast cancer cells treated with Dox, and the other two variants, full-length PTPRZ-A and PTPRZ-S, were not detected in breast cancer cells. Although our research is the first to indicate that the therapy-induced chemoresistance of breast cancer is regulated by PTPRZ1, the mechanism behind the decreases in the other splicing variants and deglycosylated isoforms that occur during chemotherapy need further exploration.

To understand the upstream mechanism of chemotherapy-driven increases in PTN expression in breast cancer, we found that the nucleoprotein CDKN1A exhibited the most upregulated expression after treatment with Dox via microarray analysis. We also found that the expression of PTN in breast cancer cells which were undergoing Dox treatment was significantly decreased when CDKN1A was knockdown. Although there is no direct evidence to clarify the mechanism of chemotherapy-driven increases in CDKN1A expression, chemotherapy may inhibit the hypermethylation of the CDKN1A promoter region, which contributes to the rescue of CDKN1A from transcriptional inactivation in breast cancer [26].

CDKN1A is well known for mediating cell cycle arrest in cancer cells [27]; however, the in-depth mechanism of CDKN1A in chemoresistance is unclear, and the role of CDKN1A in the process of chemoresistance is not consistent in different cancers. For instance, the suppressing cell growth and promoting cell cycle arrest functions of CDKN1A were observed in gastric cancer and prostate cancer [27, 28]. In contrast, we found that the expression of CDKN1A in TNBC cells was upregulated with the same trends as those observed for the expression levels of PTN and PTPRZ1 during chemotherapy, which is consistent with previous reports [29]. Moreover, we also demonstrated that increased CDKN1A expression may help TNBC cells escape cell cycle arrest during chemotherapy. It has been shown that upregulated CDKN1A expression regulates cell proliferation in TNBC cells by rescuing the inhibitory effects of liver receptor homolog-1 (LRH-1) in a p53-independent manner [29], which provides the attractive prospect that the CDKN1A/PTN/PTPRZ1 axis could be targeted for the treatment of tumours that are resistant to chemotherapy. However, we only observed the tumour-promoting effects of the CDKN1A-PTN-PTPRZ1 axis in vitro in the present study, and more evidence related to the therapeutic potential of this axis should be confirmed in vitro and in vivo in the future.

The binding of PTN to PTPRZ1 increases the tyrosine phosphorylation of downstream molecules, thus activating the signalling pathways related to cell adhesion, survival and migration [30]. We demonstrated the tumour-promoting effects of PTN in breast cancer cells undergoing chemotherapy involved the upregulation of the expression of PTPRZ1, while the increased expression of PTPRZ1 could also stimulate the secretion of PTN to form a positive feedback loop in TNBC cells. In the present study, we found that the inhibition of rhPTN on Dox could be decreased by siPTPRZ1, and the promotion of siPTN on Dox could be inhibited by overexpressed PTPRZ1. It seems that overexpression of PTPRZ1 has an effect that does not depend on PTN expression. In some other reports, PTN was found not the only activation of PTPRZ1 pathway, inflammation and immune can also activate the PTPRZ1 pathway, such as interleukin (IL)-34, an important receptor of PTPRZ1, promotes the expression of PTPRZ1 in inflammatory bowel disease (IBD) [31]. Whether other factors exist in breast cancer cells that affect the expression of PTPRZ1 under the treatment of Dox, we still need more evidence.

Further investigations of the downstream mechanism revealed that the CDKN1A/PTN/PTPRZ1 axis contributes to chemoresistance by activating the NF-κB pathway. The NF-κB family is composed of five subunits (p65/RelA, c-Rel, RelB, p105-p50/ NF-κB 1, and p100-p52/ NF-κB 2) and participates in the processes of cell cycling, immunity, angiogenesis, cell adhesion and apoptosis [32, 33]. It is well known that the activation of the NF-κB pathway depends on IKK complex activation and IκB degradation [34]. Our study suggests that Dox could significantly upregulate the expression of IKKα, p65 and p50 and induce the degradation of IKKβ and IκBα, suggesting that the classical NF-κB pathway was activated during chemotherapy. In addition, we found that the Dox + PTPRZ1 group showed more obvious activation of the NF-κB pathway than PTPRZ1 group, thus demonstrating that PTPRZ1 plays an essential role in promoting the activation of the NF-κB pathway in breast cancer cells undergoing chemotherapy. It has been shown that blocking NF-κB could restore the sensitivity of breast cancer cells to tamoxifen [35], and the possible mechanism is that the activation of the NF-κB pathway could dysregulate the apoptotic response by causing the loss of mitochondrial function and death receptor signalling, resulting in transcriptional dysregulation of apoptotic genes [36].

## Conclusions

In conclusion, we have demonstrated that the expression levels of PTN and PTPRZ1 were upregulated in breast cancer tissue after chemotherapy compared to tissue before chemotherapy. High PTN/PTPRZ1 expression is associated with poor chemosensitivity in breast cancer patients. Chemotherapy-driven increases in the CDKN1A/PTN/PTPRZ1 axis activate the NF-κB pathway in breast cancer cells. Overall, our results indicate that the chemotherapy-driven increases in the CDKN1A/PTN/PTPRZ1 axis are critical intrinsic factors of therapy-induced chemoresistance in breast cancer and that disrupting the CDKN1A/PTN/PTPRZ1 axis is a novel strategy to enhance the chemosensitivity of breast cancer, especially in the triple-negative subtype.

## Abbreviations

CDKN1A: Cyclin-dependent kinase inhibitor 1 A
DDP: Cisplatin
Dox: Doxorubicin
ER: Oestrogen receptor
GBM: Glioblastoma multiforme
LRH-1: Liver receptor homolog-1
NAC: Neoadjuvant chemotherapy
NF-κB: Nuclear factor kB
pCR: Pathologic complete response
PgR: Progesterone receptor
PTN: Pleiotrophin
PTPRZ1: Protein tyrosine phosphatase zeta
TNBC: Triple-negative breast cancer
